# Ophthalmic manifestation after SARS-CoV-2 vaccination: a case series

**DOI:** 10.1186/s12348-022-00298-y

**Published:** 2022-06-23

**Authors:** Snezhana Murgova, Georgi Balchev

**Affiliations:** Ophthalmology Department, Medical University Pleven, 91 Vladimir Vazov str, Pleven, Bulgaria

**Keywords:** COVID-19, Vaccination, Ophthalmology, Uveitis, Occlusion

## Abstract

**Background:**

The aim of this report is to describe ocular side effects in patients who received one of the two COVID-19 vaccines – Astra Zeneca or Pfizer-Biontech and to contribute to the common understanding of the COVID-19 vaccination process.

**Results:**

Three patients reactivated underlying herpetic disease and developed uveitis and keratitis. Two of them were vaccinated with Pfizer and one was with Astra Zeneca. Two patients were vaccinated with Pfizer-Biontech and had thrombosis on the 8th and 10th days following the day of vaccination. The man has diagnosed with nonarteritic anterior ischemic optic neuropathy and the woman had a subarachnoid haemorrhage, ptosis of upper eyelid and deviated eyeball.

**Conclusion:**

There is a causal relationship between vaccines and the underlying disease. For more details, further large studies are necessary.

## Background

In its quest to control the COVID-19 pandemic, humanity is undergoing the fastest and most widespread launch of vaccines in its own history [1]. For years, a link between vaccination and the occurrence of ophthalmic manifestations has been established. There is a number of post vaccination reports for influenza [2, 3], yellow fever [4], hepatitis B [5], Neisseria and other [6, 7], in which, ocular complications, such as anterior and posterior uveitis, acute maculopathy and neuroretinopathy [2, 3, 6, 7], Vogt-Koyanagi-Harada [5] and other, have been observed. This is also the case with the covid vaccines [1, 8–15]. Since Dec 2020 several COVID-19 vaccines have been launched at the world pharmaceutical market, although, our report focuses on two of them only - mRNA vaccine (BNT162b2, Pfizer-BioNTech) and vector vaccine (ChAdOx1 nCoV-19/ AZD1222, Oxford-AstraZeneca) [13, 16]. The immunological mechanism behind vaccines is extremely complex, which inevitably leads to unexpected side effects. Detailing and documenting these side effects is of particular interest to the current pandemic.

### Case 1

A 56-year-old man was admitted at the clinic with pain, photophobia and blurred vision in his right eye. Seven days prior to that, he had had second dose of Astra Zeneca COVID-19 vaccine. The history showed herpetic keratitis and iridocyclitis of the same eye a year ago. The ophthalmic examination revealed pericorneal injection, hazy cornea with paracentral thinning area dyeing with fluorescein, nongranulomatous precipitates on endothelium. Best corrected visual acuity (BCVA) was 0.02 for right eye and 1.0 for the left eye. Topical and systemic antiviral (acyclovir) therapy was started, systemic corticosteroid was added at the second stage (1 mg/kg methylprednisolon). After a month, the patient had a marked improvement with no pain or redness and BCVA of 0.7.

### Case 2

An 89-year-old lady, admitted at the clinic with blurred vision and irritation in the left eye. Three weeks prior to that, she had second dose COVID-19 vaccine of Pfizer/Biontech. The patient had an ocular history of central vein occlusion of the right eye a year ago and since then she couldn’t see well with this eye. Both eyes had cataract surgery with intraocular lens implanted years ago. She also had history for glaucoma – topically treated and underlying herpetic uveitis. At the moment of presentation, her visual acuity of the right eye was hand movement and of the left eye 0.09. Ocular pressure was 23 mmHg in the right eye and 28 mmHg in the left eye. Examination of anterior segment of both eyes showed a picture of acute anterior uveitis with mild ciliary injection, a lot of keratic nongranolomatous precipitates, aqueous flare grade 2+. Fundus examination showed cup disc ratio 0.7, old flame shaped haemorrhages on the retina in right eye. Topical and periocular (15.78 mg methylprednolsolon para-bulbar injection) corticosteroids, and antiglaucomatous therapy were administered. There was neither marked improvement during first two weeks nor aggravation of the symptoms. Patient achieved BCVA of 0.1 in the 3rd week and 0.4 in the 2nd month.

### Case 3

A 50-year-old man was referred for ophthalmology examination, because of visual disturbances in his left eye. The patient had history for underlying herpes uveitis and a few episodes of iridocyclites several months prior to that, which until that moment was in remission. Besides, he had suffered from a mild COVID-19 infection 8 month ago. The patient had had received his second dose of Pfizer-Biontech vaccine 8 days prior to ophthalmic exacerbation. On ophthalmic examination, BCVA was hand movement in the affected eye, fellow eye is 1.0. CRP and ESR are moderately elevated. No signs and symptoms from anterior segment. Moderate vitritis with exudations were present. Yellow-white lesions and haemorrhages corresponding to retinal necrosis were observed on the retina. Ultrasound reveals subtotal exudative retinal detachment. Antiviral therapy against the herpes (acyclovir) was introduced, steroids (1 mg/kg methylprednisolone) on second step, at least one day after acyclovir therapy. In the end, vitreal surgery was performed, due to enlargement of necrotic zones.

### Case 4

**Fig. 1:**
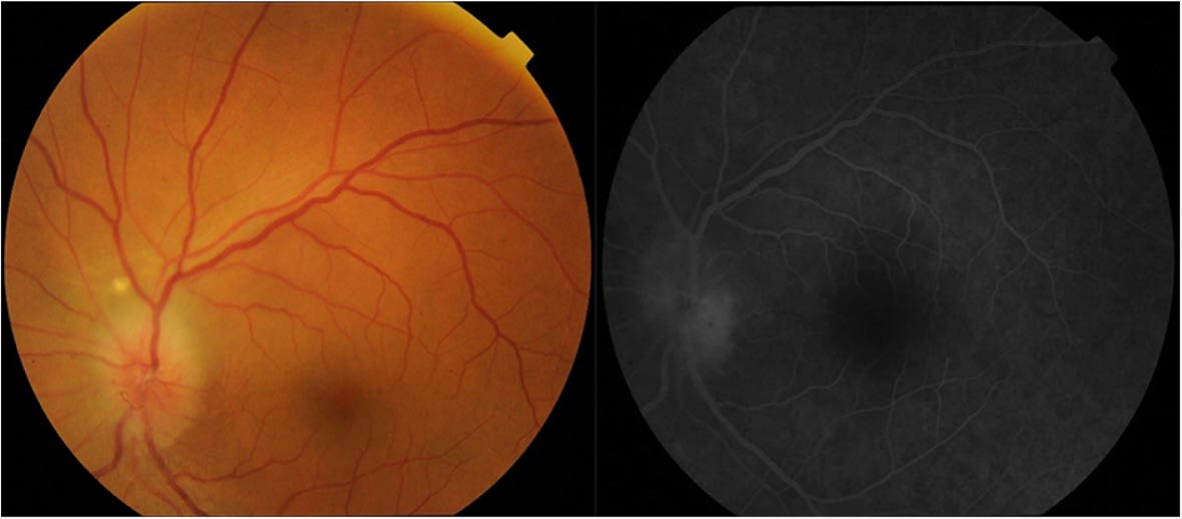
Fluorescein angiography – leakage in inferior temporal quadrant of optic disk head

A 45-year-old male was admitted at the ophthalmic clinic with complaints of visual disturbance of the left eye. Ten days prior to hospitalization, he had received second dose of Pfizer-Biontech vaccine. Medical history revealed slightly elevated blood pressure – 130/90 mmHg, cholesterol 6.8, fasting blood sugar – 6.68, normal erythrocytes sediment rate (ESR). The rest of laboratory indices were in normal range. On ocular examination, visual acuity was 1.0 in both eyes. Intraocular pressure was normal in both eyes. Fundus examination found papilloedema and slightly dilated venous vessels. Perimetry showed inferior-temporal quadrantanopia. Fluorescein angiography was performed. Patient was diagnosed with nonarteritic anterior ischemic optic neuropathy (NA-AION) (Fig. 1). Vasodilators and anti-platelet therapy were introduced. Patient BCVA was preserved to 1.0.

### Case 5

**Fig. 2:**
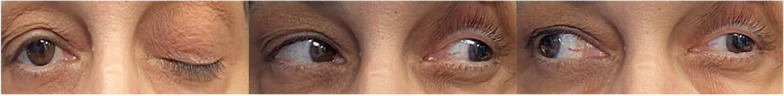
Ptosis of upper eyelid of the left eye and temporal deviated left eyeball

52-year-old female, two weeks after vaccination with Pfizer-Biontech vaccine, with headache and raised blood pressure was admitted. During the night, the headache increased and the upper eyelid of the left eye dropped. Neurologist found subarachnoid hemorrhage and surgical treatment was done. Ophthalmic examination found ptosis of the left upper eyelid (Fig. 2). Visual acuity of both eyes was 1.0. Left eye had exotropia, limited movement to upgaze, downgaze and adduction. Pupil size was 6 mm with direct reaction affected. Fundoscopy examination was normal.

## Discussion

Different post vaccination side effects have been described in the recent literature and drug’s leaflet. Thrombosis of different types [1, 9, 13, 17, 18] and exacerbation of underlying herpetic disease [8–11, 13, 19] are among the most common. The same was observed in our case series. The average age of the cited complications in the literature coincides with that of our cases (58.4 years). The time of onset of complications is similar to that of paper written by Sen and Honovar [9].

Like other authors in the literature [8], our short case series shows a reactivation of underlying herpetic uveitis and keratitis in aggravated and fulminant manner, which led to retinal detachment in one patient. That seems to be happening around the tenth day of the second dose. The mechanism behind is still unclear, but as stated from Richardson-May et al. – molecular mimicry might trigger that immune response [19].

Our clinical observation of the described cases clearly shows that patients who had received Astra Zeneca vaccine are much likely to respond to low dose corticosteroids (around 40–80 mg of methylprednisolone), unlike those, who received Pfizer-Biontech, where even high dosage of steroids (more than 80 mg of methylprednisolone) shows not sufficient results. We couldn’t find any reference in the literature concerning that statement.

The thrombosis is observed in unvaccinated patients, so it is difficult to associate them with vaccination in such small case series. However, medicine is based on a causal relationship, especially when tied to the specific time of onset and should not be easily underestimated. Thrombosis can occur in small or big vessels, due to cross-reaction of neutralizing antibodies against SARS-CoV-2 spike proteins and activated T-helper-1 cells, prior to vaccination, as described by Tsukii et al. [17]. In our series, that happened in ophthalmic branch in one patient and in subarachnoid area in another patient, as can be seen, the thrombosis may be elsewhere, although, it may manifest in the eyes.

## Conclusion

The case series presents the two SARS-CoV-2 vaccines as a possible cause for new occlusions or activation of underlying herpetic disease. Larger studies are needed to prove the relationship between the vaccines and these rare adverse effects.

## Data Availability

The datasets used and/or analysed during the current study available from the corresponding author on reasonable request.
